# Establishment and validation of a 5-factor diagnostic model for obstructive and non-obstructive azoospermia based on routine clinical parameters

**DOI:** 10.3389/ebm.2024.10137

**Published:** 2024-04-09

**Authors:** Xiaoyu Zhu, Yin Liu, Ying Huang, Hongxia Tan, Meifang He, Dong Wang

**Affiliations:** ^1^ Department of Laboratory Medicine, The First Affiliated Hospital, Sun Yat-sen University, Guangzhou, China; ^2^ Department of Laboratory Medicine, Guangxi Hospital Division of The First Affiliated Hospital, Sun Yat-sen University, Nanning, China; ^3^ Laboratory of General Surgery, The First Affiliated Hospital, Sun Yat-sen Univsersity, Guangzhou, China

**Keywords:** azoospermia, diagnostic model, follicle-stimulating hormone (FSH), seminal plasma, testicular volume

## Abstract

Azoospermia is a serious leading male-factor cause of infertility in couples of childbearing age. The two main azoospermia types, obstructive (OA) and non-obstructive (NOA) azoospermia, differ in their treatment approaches. Therefore, their clinical diagnosis is extremely important, requiring an accurate, efficient, and easy-to-use diagnostic model. This retrospective observational study included 707 patients with azoospermia treated between 2017 and 2021, 498 with OA, and 209 with NOA. Hematological and seminal plasma parameters, hormone levels, and testicular volume were used in logistic regression analysis to evaluate and compare their diagnostic performance, results showed that the optimal diagnostic model is constructed by five variables including semen volume, semen pH, seminal plasma neutral α-glucosidase activity, follicle-stimulating hormone in the serum, and testicular volume, compared with follicle-stimulating hormone-based and testicular volume-based models. The 5-factor diagnostic model had an accuracy of 90.4%, sensitivity of 96.4%, positive predictive value of 90.6%, negative predictive value of 89.8%, and area under the curve of 0.931, all higher than in the other two models. However, its specificity (76.1%) was slightly lower than in the other models. Meantime, the internal 5-fold cross-validation results indicated that the 5-factor diagnostic model had a good clinical application value. This study established an accurate, efficient, and relatively accessible 5-factor diagnostic model for OA and NOA, providing a reference for clinical decision-making when selecting an appropriate treatment.

## Impact statement

At present, the incidence of male infertility is gradually increasing, and many couples of childbearing age suffer from it. Among them, azoospermia is one of the most common and serious causes of male infertility, mainly divided into obstructive azoospermia and non-obstructive azoospermia. The optimal treatment options for the two types of azoospermia are different, so clinically accurate and efficient differentiation is required. In this study, a highly accurate diagnostic model was established using clinical routine parameters such as semen volume, semen pH, seminal plasma neutral α-glucosidase activity and follicle-stimulating hormone, as well as testicular volume. Compared with the traditional gold standard “testicular biopsy,” this model is simple, non-invasive, efficient and accurate, which can provide a good reference for clinical decision-making, and its practical application value needs to be tested in a larger population.

## Introduction

The incidence of male infertility is increasing. It has been reported that about 10–15% of all couples of reproductive age face fertility-related problems, and about 50–60% of these can be attributed to males [1–3]. Azoospermia is a common and the most severe cause of male infertility, affecting approximately 10–15% of infertile males [4–6]. Azoospermia means that no spermatozoa are found in two or more ejaculated semen samples [6]. Azoospermia is divided into obstructive (OA) and non-obstructive (NOA) azoospermia types. In OA, spermatogenesis is normal, but the sperm cannot be excreted normally due to mechanical obstruction along the reproductive tract, including the vas deferens, epididymis, and ejaculatory ducts. The spermatogenic dysfunction of NOA is associated with inherent defects in the testes caused by various factors that severely affect the process, usually resulting in primary testicular failure or dysfunction of the hypothalamic-pituitary-gonadal axis [4, 6].

Advances in sperm retrieval and microsurgical techniques make it possible for more couples with infertility to have progeny. The two azoospermia types are clinically treated differently to achieve the best therapeutic effect. Patients with OA are often referred for surgical removal of obstruction, while those with NOA may require assisted reproductive technologies, including microdissection testicular sperm extraction [7]. Particularly, in some cases, in addition to conventional microsurgical reconstruction operations such as vasoepididymistomy, patients with epididymis obstruction or inguinal vessel obstruction can also be offered percutaneous epididymal sperm aspiration (PESA), microsurgical epididymal sperm aspiration (MESA), or testicular sperm aspiration (TESA), and patients with intratesticular obstruction can take conventional testicular sperm extraction (TESE) [3, 8]. Therefore, an accurate diagnosis of the azoospermia type is very important for clinical treatment selection. A comprehensive clinical assessment is made to clarify the azoospermia type, comprising the results of medical history, physical examination, semen analysis, hormone evaluation, genetic testing, and various imaging examinations. These can lead to a definite diagnosis in over 90% of the patients with azoospermia; however, this comprehensive evaluation is time- and workforce-demanding, significantly increasing the diagnostic and treatment costs. Furthermore, a definite diagnosis cannot be reached for some patients, requiring testicular biopsy, the gold standard for OA and NOA diagnosis. However, a biopsy might cause testicular damage, so the relevant guidelines do not recommend using this method as a routine diagnostic tool to determine the azoospermia type [4]. An accurate and efficient clinical diagnostic model that can reduce the diagnosis and treatment costs for patients with azoospermia; however, such diagnostic models of azoospermia are scarce and limited. Studies have shown that males with elevated serum follicle-stimulating hormone (FSH) and reduced testosterone/FSH ratio are more likely to have abnormal semen analysis [9], reflecting lesions in the male genitourinary system [10]. The FSH-based model established by Tradewell et al. was good at predicting the probability of azoospermia but could not be used for differential diagnosis [11].

This study aimed to construct a clinical diagnostic model to differentiate between OA and NOA using conventional clinical parameters such as testicular volume, seminal plasma composition, and hormone levels. The receiver operating characteristic (ROC) curve was used to assess the diagnostic ability of the developed models and identify the optimal one. Such a model would be a valuable clinical tool for azoospermia diagnosis, ultimately providing a preliminary reference for selecting appropriate clinical treatment methods.

## Materials and methods

### Study population

This retrospective study included 707 patients with azoospermia treated at the First Affiliated Hospital of Sun Yat-Sen University between 2017 and 2021, 498 with OA, and 209 with NOA. All patients were diagnosed by ultrasonographic examination of the urogenital system and testicular biopsy. Among them, 493 patients underwent testicular biopsy in dedicated Fertility Center Laboratory of the hospital. According to histopathological results, it was diagnosed as OA if spermatogenic cells and mature spermatozoa at all levels can be seen in the seminiferous tubules, and the number is generally normal or slightly reduced, and/or there is obstruction or absence of the epididymis, vas deferens, or ejaculatory ducts according to the ultrasound results; while the presence of markedly reduced testicular volume, cryptorchidism, or testicular parenchymal lesions was diagnosed as NOA. However, most patients were diagnosed as NOA when biopsy results showed that all levels of spermatogenic cells with a small amount of mature spermatozoa could be seen in the seminiferous tubules, significantly reduced mature spermatozoa or even azoospermia or Sertoli cell-only syndrome, or ultrasonography showed substantial changes in the testicles, and only a few patients was diagnosed as OA as the ultrasound results showed obstruction, semen stasis, or reproductive duct deficiency. The remaining 214 patients did not undergo testicular biopsy, and the diagnosis was mainly based on clinical manifestations and ultrasound results, including whether there was obstruction, absence of reproductive tract, and testicular volume, et al. During the testicular biopsy, the laboratory will immediately cryopreserve all the viable sperm if any of them are extracted. Patients with liver, kidney, hypothalamic-pituitary, and other reproductive system diseases were excluded.

### Data collection

The following data were retrieved from the institute’s database to construct the models: age; hematological parameters, including absolute values of leukocytes, neutrophils, monocytes, lymphocytes, eosinophils, basophils, red blood cells (RBC), and platelets, mean corpuscular volume, and hemoglobin; semen and seminal plasma analysis, including semen volume, pH, seminal fructose and elastase concentrations, seminal plasma neutral α-glucoside activity (SPNG); serum levels of FSH, luteinizing hormone (LH), testosterone, progesterone, estradiol, and prolactin; testicular volume.

### Establishment and evaluation of the model

The OA and NOA groups were compared using nonparametric tests to screen for significantly different parameters. The parameters selected for inclusion in the initial logistic regression model were RBC, hemoglobin, platelets, semen volume, semen pH, seminal fructose, SPNG, FSH, LH, testosterone, prolactin, and testicular volume. Independent variables with a significant (*p* < 0.05) impact on the outcome were further screened to establish possible diagnostic models. Finally, we used the area under the ROC curve (AUC) to calculate the accuracy, sensitivity, specificity, positive predictive value (PPV), and negative predictive value (NPV) of the models. The diagnostic performance of the models was evaluated (see below), and the optimal one was selected.

### Internal validation of the model

The internal validation of the model tested its generalization ability and evaluated its clinical application value. We used the machine learning tool in the DxAI intelligent scientific research platform (Deepwise, Beijing, China) with a 5-fold cross-validation method to validate the model. This validation method randomly divides all the research subjects into five average groups, using four groups as a training set and the fifth as a validation set. This process was repeated, using each time a different group as the validation set. The average value of the five evaluation results was used as the final evaluation indicator. The assessed metrics included the AUC, accuracy, sensitivity, specificity, PPV, and NPV.

### Statistical analysis

This study used IBM SPSS Statistics for Windows, Version 20.0 (IBM Corp., Armonk, NY, United States) for data analysis. The Kolmogorov-Smirnov test assessed whether the variables were normally distributed. Continuous variables are described as medians (interquartile ranges), and categorical variables as frequencies (percentages). The Kolmogorov-Smirnov test compared continuous variables between two independent samples, and the Mann-Whitney U test compared categorical variables. Missing data values were completed using the expectation-maximization model. Binary logistic regression was used for multivariate analysis to obtain the corresponding diagnostic model. The stepwise regression method (forward-likelihood ratio) was used to screen independent variables, that is, the independent variables are introduced into the regression equation one by one, and the models with and without an independent variable are compared by the likelihood ratio statistic, and if there is statistical significance, the independent variable is included in the model, and *vice versa*, until no independent variable can be introduced. The models’ ability to differentiate OA from NOA was evaluated using ROC analysis, and the MedCalc, Version 20.115 (MedCalc Software Ltd., Ostend, Belgium) was used for comparison of different ROC curves. The machine learning process was carried out on the DxAI intelligent scientific research platform. All tests were two-sided, and the significance level was set at *p* < 0.05.

## Results

### Demographic and clinical characteristics of the research subjects

During the study period, 707 patients (median age is 30, IQR is 6) with azoospermia were admitted to the First Affiliated Hospital of Sun Yat-sen University, 209 with NOA and 498 with OA. The clinical characteristics, hematological parameters, semen and seminal plasma parameters, serum hormone levels, and testicular volume of the two groups are summarized in Table 1. The analysis results showed that the RBC, hemoglobin, and testosterone in the OA group were significantly higher (*p* < 0.01) and FSH, LH, and prolactin significantly lower (*p* < 0.01) than in the NOA group. The groups also differed significantly in testicular volume (*p* < 0.01). Based on a normal testicular volume of 12–19 mL [13], 88.5% of the patients in the NOA group had unilateral or bilateral testicular volume reduction or cryptorchidism, while this was found in only 46.6% of the patients in the OA group. Nearly half (44.6%) of the OA group had normal testicular volume compared with 10.5% of the NOA group. Other indicators, such as age, the total number of leukocytes and their components, mean corpuscular volume, seminal plasma elastase, progesterone, and estradiol levels, were similar in both groups.

**TABLE 1:** Demographic and clinical characteristics of OA and NOA groups.

Variable	NOA (*n* = 209)	OA (*n* = 498)	*p*-value
Age (years)	30.00 (6.00)	30.00 (7.00)	0.72
Leukocytes (×10^9^/L)	6.93 (2.39)	6.98 (2.02)	0.75
Neutrophils (×10^9^/L)	3.99 (1.89)	4.00 (1.69)	0.55
Monocytes (×10^9^/L)	0.49 (0.18)	0.49 (0.20)	0.98
Lymphocytes (×10^9^/L)	2.16 (0.75)	2.19 (0.79)	0.99
Eosinophils (×10^9^/L)	0.12 (0.14)	0.13 (0.17)	0.16
Basophils (×10^9^/L)	0.03 (0.03)	0.03 (0.03)	0.85
RBC (×10^12^/L)	5.16 (0.64)	5.28 (0.54)	**0.002** **
MCV (fL)	88.45 (5.27)	88.80 (5.80)	0.29
Hemoglobin (g/L)	152.00 (14.00)	157.00 (13.50)	**0.000** **
Platelets (×10^9^/L)	259.00 (79.50)	241.00 (71.00)	**0.002** **
Semen volume (mL)	3.50 (2.00)	1.60 (1.70)	**0.000** **
Semen pH	7.50 (0.00)	7.50 (1.00)	**0.000** **
Seminal plasma fructose (µmol)	28.90 (43.85)	2.90 (16.25)	**0.000** **
SPNG (mU)	24.80 (39.75)	2.90 (8.35)	**0.000** **
Seminal plasma elastase (ng/mL)	169.20 (472.35)	205.80 (827.85)	0.14
FSH (IU/L)	14.69 (13.05)	3.67 (2.32)	**0.000** **
LH (IU/L)	5.08 (3.58)	2.84 (1.69)	**0.000** **
Testosterone (ng/mL)	3.97 (2.81)	4.89 (2.69)	**0.000** **
Progesterone (ng/mL)	0.30 (0.20)	0.30 (0.10)	0.72
Estradiol (pg/mL)	21.00 (13.00)	21.00 (12.00)	0.96
Prolactin (ng/mL)	12.13 (10.55)	10.35 (6.04)	**0.000** **
Testicular size			
Bilateral small testes or cryptorchidism	172 (82.3%)	142 (28.5%)	**0.000** **
Unilateral small testis or absence/cryptorchidism	13 (6.2%)	90 (18.1%)	
Bilateral normal testicular volume	22 (10.5%)	222 (44.6%)	
Unilateral or bilateral large testicular volume	2 (1.0%)	44 (8.8%)	

**p < 0.01. OA, obstructive azoospermia; NOA, non-obstructive azoospermia; SPNG, seminal plasma neutral α-glucosidase activity; FSH, follicle-stimulating hormone; RBC, red blood cells; LH, luteinizing hormone; MCV, mean corpuscular volume.

The bold values mean significant different variable between OA and NOA.

### Establishment and evaluation of the model

**FIGURE 1:**
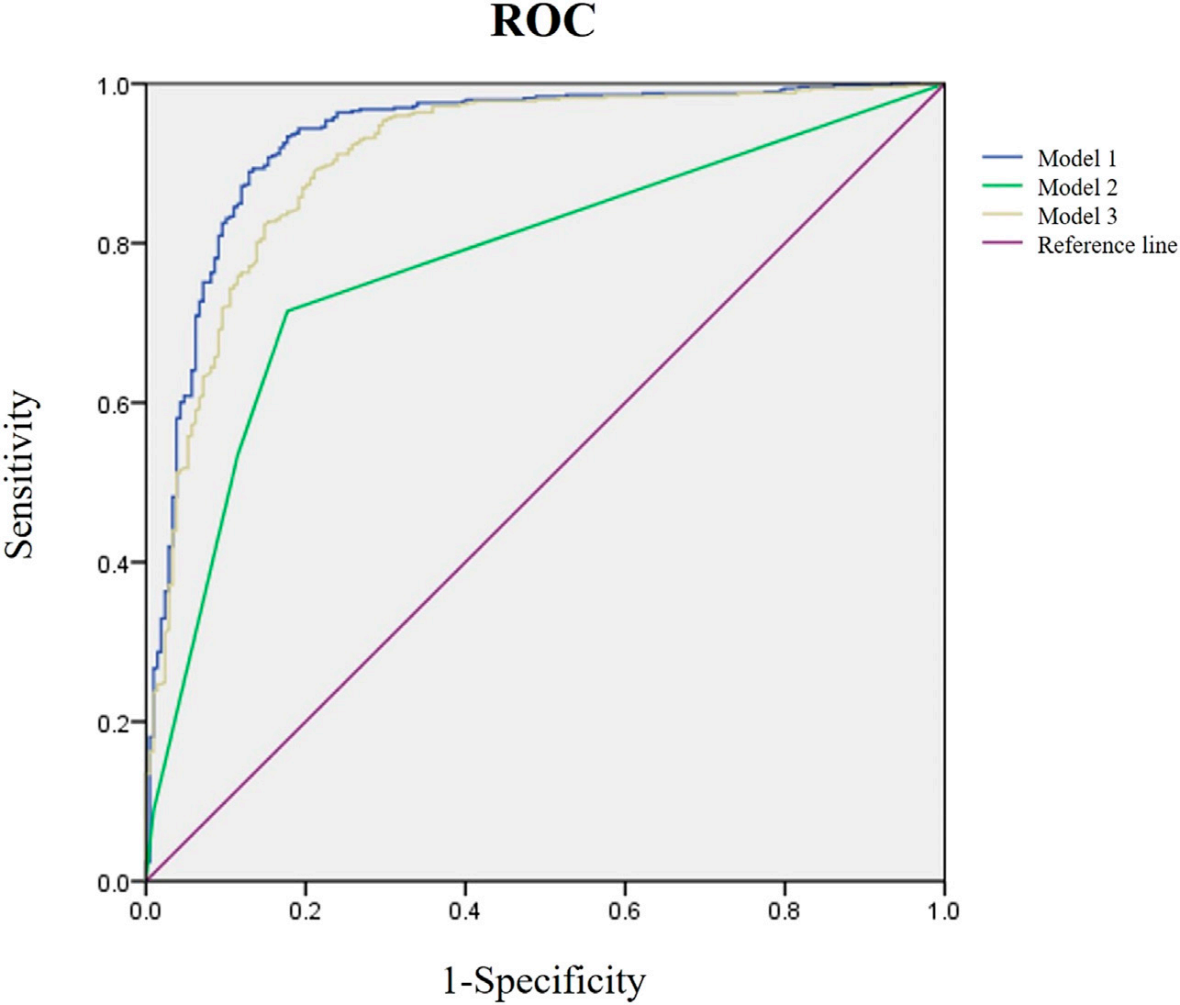
Receiver operating characteristic (ROC) analysis of three diagnostic models. The purple line is the reference, the blue is Model 1, the green is Model 2, and the yellow is Model 3.

We included indicators significantly different between the OA and NOA groups as candidates for the logistic regression analysis and screened independent variables with the stepwise regression method (forward-LR) to avoid including too many independent variables and minimize the influence of irrelevant ones, improving the diagnostic model performance. Finally, a diagnostic model was established with five variables, including semen volume, semen pH, SPNG, FSH, and testicular volume (Model 1). Since the testicular volume had the greatest impact on the diagnostic results in the logistic regression model (odds ratio, 0.013; 95% confidence interval, 0.000–0.457, *p* = 0.017), it was used as an independent risk factor to establish a new diagnostic model (Model 2). Furthermore, FSH has been used alone to predict azoospermia [11] and as a diagnostic criterion for NOA [6, 12]. Therefore, we used FSH as an independent risk factor to establish another diagnostic model (Model 3). The logistic regression analysis results of the three models are shown in Table 2, and their ROC curves are shown in Figure 1. The three models were compared for accuracy, sensitivity, specificity, PPV, NPV, and AUC (in Table 3), showing that the diagnosis accuracy of Model 1 (90.4%) was higher than that of Models 2 (74.7%) and 3 (87.1%). The AUC of Model 1 (0.931) was significantly larger than that of Model 2 (0.778, *p* < 0.01) and Model 3 (0.908, *p* < 0.01).

**TABLE 2:** Three diagnostic model types to differentiate OA from NOA with their independent variables.

Model	Variable	B	*p*-value	OR (95% CI)
Model 1	Constant	15.131	0.000**	3726840.721
	Semen volume	−0.263	0.002**	0.769 (0.652–0.907)
	Semen pH	−1.090	0.003**	0.336 (0.164–0.690)
	SPNG	−0.015	0.002**	0.986 (0.977–0.994)
	FSH	−0.192	0.000**	0.826 (0.785–0.868)
	Testicular size		0.000**	
	Testicular size (1)	−4.362	0.017*	0.013 (0.000–0.457)
	Testicular size (2)	−2.918	0.116	0.054 (0.001–2.055)
	Testicular size (3)	−2.861	0.121	0.057 (0.002–2.138)
Model 2	Constant	3.091	0.000**	22.000
	Testicular size		0.000**	
	Testicular size (1)	−3.283	0.000**	0.038 (0.009–0.157)
	Testicular size (2)	−1.156	0.139	0.315 (0.068–1.456)
	Testicular size (3)	−0.779	0.303	0.459 (0.104–2.021)
Model 3	Constant	3.326	0.000**	27.814
	FSH	−0.308	0.000**	0.735 (0.699–0.773)

*p < 0.05, **p < 0.01. Note: The testicular size was used as an ordered multi-categorical variable. It was assigned a value of 1 for bilateral small testes or cryptorchidism, 2 for unilateral small testis or absence/cryptorchidism, 3 for bilateral normal testicular volume, and 4 for unilateral or bilateral large testicular volume.

OA, obstructive azoospermia; NOA, non-obstructive azoospermia; SPNG, seminal plasma neutral α-glucosidase activity; FSH, follicle-stimulating hormone; OR, odds ratio; CI, confidence interval.

The bold values mean independent risk factors in each diagnostic model screened through the logistic regression analysis.

**TABLE 3:** Efficacy evaluation of three diagnostic models for OA and NOA.

Models	Accuracy (%)	Sensitivity (%)	Specificity (%)	PPV (%)	NPV (%)	AUC
Model 1	90.4	96.4	76.1	90.6	89.8	0.931
Model 2	74.7	71.5	82.3	90.6	54.8	0.778
Model 3	87.1	96.8	64.1	86.5	89.3	0.908

OA, obstructive azoospermia; NOA, non-obstructive azoospermia; PPV, positive predictive value; NPV, negative predictive value; AUC, area under the receiver operating characteristic curve.

### Model verification

**FIGURE 2:**
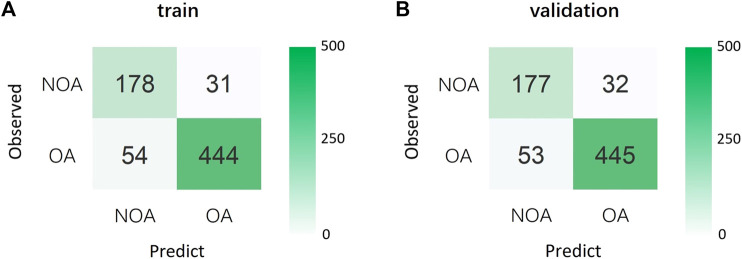
**(A)** Confusion matrix for the training set. **(B)** Confusion matrix for the validation set. OA, obstructive azoospermia; NOA, non-obstructive azoospermia.

**FIGURE 3:**
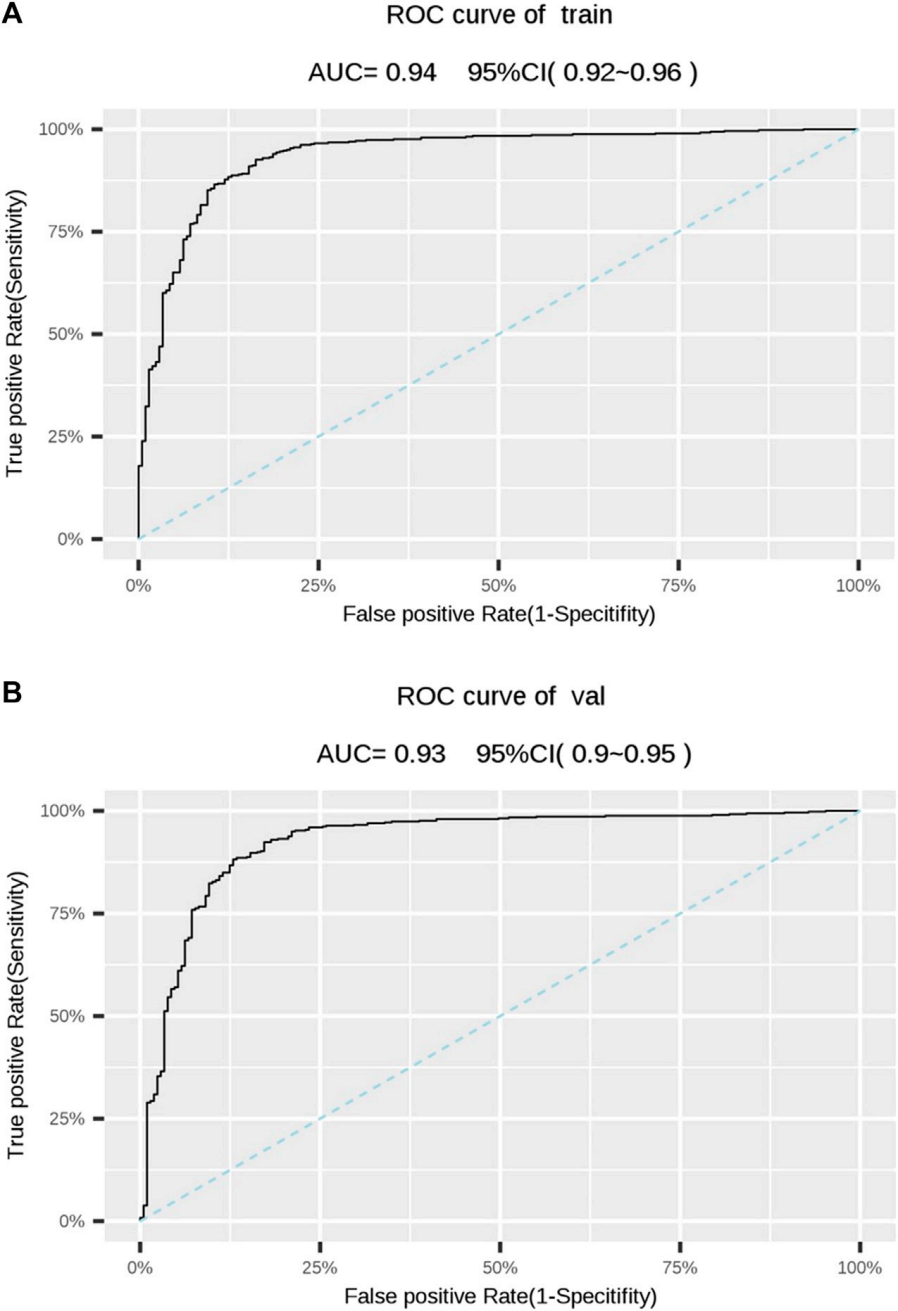
**(A)** Receiver operating characteristic (ROC) of the training set. **(B)** ROC of the validation set. The dotted line represents is the reference. AUC, area under the ROC curve; CI, confidence interval.

We conducted a 5-fold cross-validation of Model 1 to verify its generalization ability and evaluate the effectiveness of its diagnostic performance (Figure 2). The results showed that the training and validation sets had an accuracy of 88.0%. Moreover, its sensitivity, specificity, and PPV were all above 80%; however, the NPV was slightly lower than in the other two models. Notably, the AUCs of the training and validation sets were 0.938 and 0.928, respectively, indicating that Model 1 could accurately distinguish OA from NOA (Table 4; Figures 3A, B).

**TABLE 4:** Internal 5-fold cross-validation results for Model 1.

Set	Accuracy (%)	Sensitivity (%)	Specificity (%)	PPV (%)	NPV (%)	AUC
Training	88.0	89.2	85.2	93.5	76.7	0.938
Validation	88.0	89.4	84.7	93.3	77.0	0.928

PPV, positive predictive value; NPV, negative predictive value; AUC, area under the receiver operating characteristic curve.

## Discussion

This study screened clinical, hematological, and seminal plasma parameters, hormone levels, and testicular volume in patients with OA and NOA to construct diagnostic models. The selected diagnostic model to distinguish OA from NOA included semen volume, semen pH, SPNG, FSH, and testicular volume (AUC of 0.931). We used cross-validation to confirm the clinical diagnostic validity of this model.

Routine hematological parameters are indispensable in clinical disease screening as they reflect the basic physiological and pathological conditions of the individual. In this study, only the RBC, hemoglobin, and platelets differed significantly between the OA and NOA groups. However, these parameters had an insignificant effect on the diagnostic outcome in the logistic regression analysis, indicating that differentiation between the two azoospermia types could not be performed accurately based on routine blood tests. However, the construction of a diagnostic model for OA and NOA was urgently needed to distinguish between various azoospermia types.

The parameters included in the model (seminal plasma parameters, hormone levels, and testicular volume) were assessed in studies on azoospermia. Studies demonstrated that the ejaculation volume of azoospermia patients with normal sperm motility was significantly smaller than that of patients with inactive spermatozoa [13]. Furthermore, serum levels of LH, testosterone, FSH, progesterone, estradiol, and other hormones affect spermatogenesis, especially FSH, which plays an important role in Sertoli cells and has an important regulatory effect on sperm formation [14]. A study by Muttukrishna et al. showed that FSH could distinguish OA from NOA, as it is usually normal or slightly elevated in patients with OA and significantly elevated in patients with NOA [15]. The testicular volume can be measured by non-invasive ultrasonographic methods and is closely related to testicular function [16, 17]. Our results were consistent with a previous study that found that patients with NOA had significantly smaller testicles than those in the OA and healthy control groups [18]. Another research showed that patients with severe testicular histopathology had higher serum FSH levels and smaller testicles in a NOA population [19]. The final diagnostic model that included 5 routine clinical parameters was more reliable for differential diagnosis of azoospermia than models based on a single factor (FSH or testicular volume).

Several studies reported diagnostic models to tell OA and NOA apart, using ultrasonography-derived testicular volume alone or in combination with abnormal vas deferens, FSH, or ultrasonography-related parameters. However, these studies assessed relatively few subjects and did not conduct model validation [7, 20–22]. Recent studies focused on omics methods for disease biomarker screening, including studies that screened potential biomarkers of NOA by transcriptome analysis of the testicular tissue [23, 24]; similarly, some researchers used testicular biopsy proteomic analysis to screen possible protein markers and pathways that could distinguish OA from NOA. Ritesh et al. screened seminal plasma mRNA to differentiate OA and NOA [25]. These studies identified potential biomarkers for diagnosing OA and NOA, but these biomarkers should be validated in an in-depth clinical study. Furthermore, although testicular biopsy is the gold standard for azoospermia diagnosis, it is not recommended as a routine clinical diagnostic procedure due to its invasiveness and the possibility of missing the spermatogenic regions [25, 26]. In addition, testicular biopsy may be damaging to the few remaining spermatogenesis sites in NOA patients and should be considered only in cases where no other diagnostic modalities can be used to obtain definitive results. At the same time, it should be carried out in a professional reproductive laboratory, so that all extracted active sperm can be cryopreserved in time. A multicenter study of the developed diagnostic model is needed before applying it clinically.

In conclusion, this study established and validated a 5-parameter clinical diagnostic model to distinguish OA from NOA using logistic regression analysis. The model included semen volume, semen pH, SPNG, FSH, and testicular volume. This model provides a new method to tell OA and NOA apart and acts as a reference for instituting clinical treatment and other potential clinical applications.

## Data Availability

The original contributions presented in the study are included in the article/supplementary material, further inquiries can be directed to the corresponding authors.
